# Longitudinal Monitoring of Abscess Microbiota in a Wild California Sea Lion (*Zalophus californianus*) Using Next-Generation Sequencing

**DOI:** 10.3390/ani16101498

**Published:** 2026-05-13

**Authors:** Jenna Archambeau, Lauren Palmer, Megan Wier, Nathan Sharp, Janina Krumbeck

**Affiliations:** 1 MiDOG Animal Diagnostics, Tustin, CA 92780, USA; jarchambeau@midogtest.com; 2 Marine Mammal Care Center Los Angeles, San Pedro, CA 90731, USA; lpalmer333@aol.com (L.P.); mwier@marinemammalcare.org (M.W.); 3 Perris Animal Hospital, Perris, CA 92570, USA; dvmsharp@gmail.com

**Keywords:** cervical neck abscess, California sea lion, pathogen, microbial infection, next-generation sequencing, antibiotic stewardship, *Fusobacterium gonidiaformans*

## Abstract

Antimicrobial resistance is a growing threat to human and animal health and is recognized as a global One Health issue. Strategies to reduce its impact include judicious usage of antibiotics guided by accurate bacterial identification and antimicrobial susceptibility. Next-generation sequencing (NGS) offers a DNA-based diagnostic approach that profiles the bacteria microbiota and can help support more informed antibiotic selection. We utilized NGS to identify *Fusobacterium gonidiaformans* in the neck abscess of a wild California sea lion (*Zalophus californianus*) undergoing rehabilitation. Initially, NGS revealed that the microbial population was dominated by *F. gonidiaformans*. Following treatment, the microbial profile shifted over the following eight weeks, during which 13 other bacterial species were detected, demonstrating the need for repeated testing to monitor changes in persistent lesions. Despite effective treatment to eliminate *F. gonidiaformans* from the abscess, the patient died during treatment due to suspected unrelated causes. Necropsy and histopathology identified bacterial pneumonia and sepsis associated with a large squamous cell carcinoma of the oropharynx and neck, all contributing to a deadly outcome. To our knowledge, this is the first case report of the use of longitudinal NGS sampling with the identification of Fusobacterium gonidiaformans associated with an abscess in the neck in a California sea lion. These findings support the utility of NGS in monitoring microbial dynamics to allow informed decision-making in antimicrobial selection in complex infections.

## Simple Summary

This case report describes the use of next-generation DNA sequencing (NGS) to characterize microbial changes in a cervical abscess in a rehabilitating wild California sea lion *(Zalophus californianus*). Initial sequencing revealed dominance by the anaerobic Gram-negative bacillus *Fusobacterium gonidiaformans*. Following targeted treatment of the microorganism, microbial diversity initially decreased, then increased with other bacterial species emerging after elimination of the primary bacterial pathogen. Despite successful treatment targeting *Fusobacterium gonidiaformans*, the animal succumbed to an unrelated bacterial pneumonia revealed at necropsy and confirmed by histopathology. To our knowledge, this case is the first documented report of *F. gonidiaformans* associated with a cervical neck abscess in a California sea lion and highlights the clinical utility of NGS for identifying pathogens in complex microbial infections in wildlife.

## 1. Introduction

The increasing prevalence of antimicrobial resistance threatens health outcomes for humans and animals and is recognized as a global One Health issue [1,2,3]. Multidrug-resistant (MDR) bacteria now pose global threats to human and domestic animal health by evading antibiotic treatments and limiting treatment options [4,5,6]. The mortality associated with antimicrobial resistance genes (AMRs) is projected to increase to 10 million people in the next 25 years [7]. To slow the spread of AMRs and to preserve the efficacy of antibiotics, recommendations for the stewardship and judicious use of antibiotics by veterinary professionals are driving a need for the timely identification of bacterial pathogens with indications for appropriate antibiotic selection and use [8,9].

Wildlife rehabilitation of abandoned, sick, injured, or malnourished animals, with intent to return them to their natural environment, poses a specific risk of AMR spread. In addition to human and domestic animal exposure to AMRs, reports in wildlife, with and without proximity to anthropogenic sources, are also increasing in avians and in terrestrial and marine mammals [10,11,12,13,14]. Reports of stranded marine mammals admitted to a rehabilitation hospital for abscesses and umbilical infections describe patients commonly colonized by MDR bacteria prior to treatment [15]. Moreover, the risk of exposure to MDR bacteria in rehabilitation is enhanced when a large number of animals, with a variety of ailments, are treated with antimicrobials and are held in close proximity for extended periods. In a study of stranded juvenile elephant seals, fecal testing upon admission and again at release demonstrated an increase in AMR bacteria regardless of whether animals had been treated with antibiotics [10]. In addition to these exposures, wild animals often have a limited tolerance to antimicrobial therapy or have limited routes by which antibiotics can be administered, further reducing the number and type of antibiotics available to the rehabilitator. Antimicrobial selection is frequently based on empirical judgement or effective use in other species.

Next-generation sequencing (NGS) is a DNA-based diagnostic method that supports an evidence-based approach to antibiotic selection by measuring the entire bacterial microbiota. NGS analyses are expanding our understanding of polymicrobial communities and the genes that encode for natural and acquired mechanisms of antibiotic resistance [16]. With the development of NGS, the specific bacterial DNA within a sample can be used to identify all microbes present by comparing the sample DNA to a reference library of genetically known microbes, including rare pathogens that would otherwise not be detectable with traditional culture methods [17,18]. One advantage of NGS for exotic or wild animal species is that many bacteria inhabiting these animals are difficult to culture due to their fastidious nature. 

While NGS has been widely applied in human and domestic animal medicine, its use in wildlife, particularly marine mammals, remains limited. Abscesses in sea lions are often polymicrobial, yet longitudinal microbial monitoring has rarely been reported. Repeated sampling for NGS offers the clinician the opportunity to identify and monitor the abundance of all bacterial microbes present so that antibiotic selection can be tailored to both dominant and mixed bacterial populations as the composition undergoes change [19]. As described in this case report, the bacterial microbiota of a large neck abscess in a sea lion was measured over eight weeks using NGS.

## 2. Case Signalment

**Figure 1:**
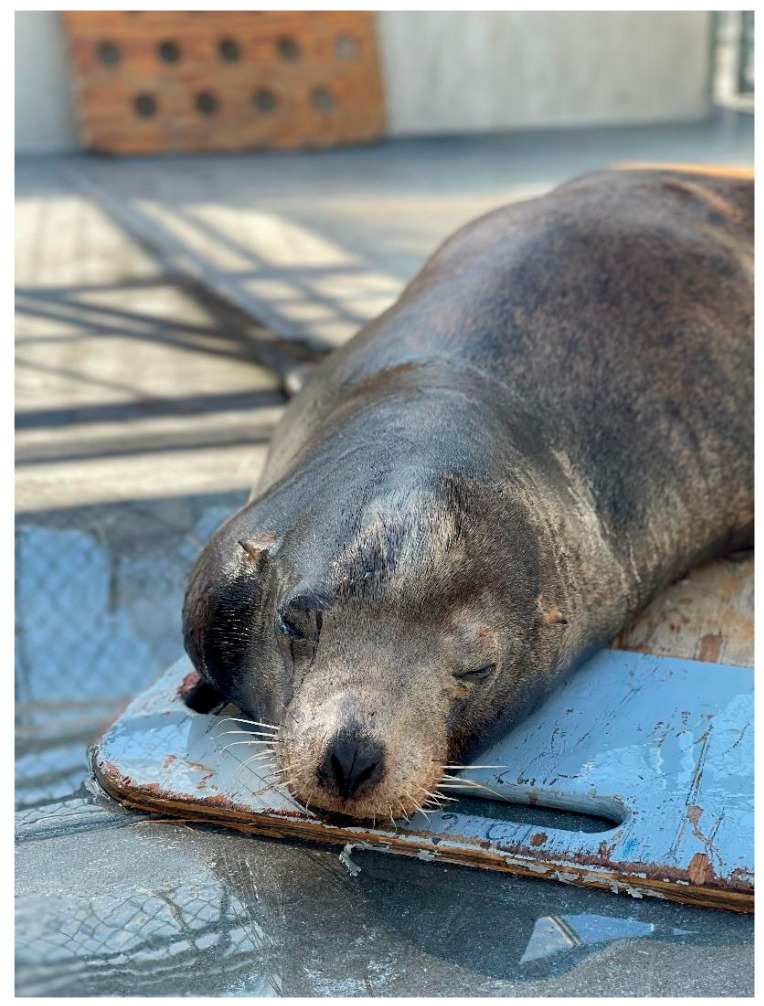
Adult male California sea lion with right neck abscess; patient is under sedation.

An adult, male, California sea lion was observed in Marina del Rey, CA (Latitude 33.972886, Longitude −118.453142) on 11 January 2023 with a large mass on the right side of his neck that inhibited the cervical range of motion. He was successfully rescued six days later (17 January 2023) and admitted to Marine Mammal Care Center Los Angeles for care (Figure 1). The patient weighed 157 kg and was 222 cm in length, and the circular-shaped neck mass measured approximately 14 × 14 × 15 cm. The patient had a body condition score of 2/5 and was mildly dehydrated at 5%.

## 3. Case Management

### 3.1. Sedation

Upon admission, the patient was sedated with Dexdomitor^®^ (dexmedetomidine hydrochloride, Zoetis Parsippany, NJ, USA, 0.013 mg/kg) and midazolam (0.13 mg/kg). The patient was sedated weekly over the ensuing eight weeks using either this protocol or medetomidine/vatinoxan hydrochloride (0.013–0.015 mg/kg) (Zenalpha^®^ Dechra, Overland Park, KS, USA) and midazolam (0.15 mg/kg). In most cases, a large animal squeeze cage was used for restraint to facilitate intramuscular injections. Isoflurane was administered as needed by mask or endotracheal intubation. Reversal of Dexdormitor or Zenalpha was achieved with Antisedan^®^ (atipamezole hydrochloride, Zoetis Parsippany, NJ, USA), and midazolam was not reversed.

### 3.2. Sampling

The abscess was multiloculated, which inhibited flushing and drainage through stab incisions; however, approximately 700 mls of tan, purulent fluid was recovered through 19-gauge 1 ½” and 20-gauge 2” needles at each sampling. Swabs for NGS testing and blood for a complete blood count (CBC) and serum biochemical (CHEM) profile were collected on each of the following dates: 17 January 2023, 23 January 2023, 26 January 2023, 2 January 2023, 9 February 2023, 16 February 2023, 3 March 2023, 17 March 2023, 21 March 2023. Additionally, samples collected on 9 February 2023 and 16 February 2023 were submitted for cytology. On 17 March 2023 an exploratory surgery of the mass with drainage, debridement and flushing was done. During this procedure a mass and open wound in the left side of the oropharynx (opposite from the side of the abscess) were discovered. Laboratory culture in addition to NGS bacterial identification and histopathology was performed on samples from the neck and oral lesion.

### 3.3. Treatment

Upon admission the patient was given a single subcutaneous injection of Baytril^®^ 100 Enrofloxacin (Elanco US Inc., Shawnee, KS, USA, 5.9 mg/kg). This antibiotic was selected for its broad spectrum, bactericidal activity, and subcutaneous absorption. Other treatments and diagnostics included three liters of Vetivex^®^ Lactated Ringers Injection, USP (Dechra, Overland Park, KS, USA) and radiographs of the abscess to rule out foreign bodies as a source of infection. The thickness of the patient’s neck made imaging challenging, and only the abscess was successfully imaged; no foreign bodies were detected. Ultrasonography was not available. The patient began eating fish the first day, and oral administration of antibiotics was elected thereafter. Clindamycin (11.4 mg/kg PO q 12) was initiated on 18 January 2023 and doxycycline (10 mg/kg PO q 24) on 1/19/2023. These antibiotics were given empirically as abscesses in sea lions often maintain both aerobic and anaerobic bacteria [20,21]. Doxycycline was discontinued after five days when NGS results indicated that the only bacteria identified, *F. gonidaformans*, should respond to clindamycin. In addition to *F. gonidiaformans* being identified, five species of fungi at cell counts between 1 and 7 were present; treatment was not pursued due to the low cell counts. The cutaneous nature of the abscess caused us to add amoxicillin (22 mg/kg PO q 12) on 25 January 2023 in the event that sampling of the multiloculated mass had missed commonly encountered Gram-positive skin bacteria such as *Staphylococcus* spp. or *Streptococcus* spp. These two antibiotics were continued for the next four weeks and were discontinued on 28 February 2023 when cell counts for *F. gonidiaformans* were almost zero. The patient was housed alone in a dry area for the duration of his rehabilitation.

### 3.4. Clinical Outcomes

Initially, the patient appeared bright, responded to his surroundings, ate well and gained 29.4 kg until 15 March 2023 when his appetite waned. On 21 March 2023, four days after the last sedation, during which a mass was discovered in the oral cavity, he vomited a rusty red-colored fluid, became ataxic and exhibited labored breathing. He was found deceased shortly thereafter. A necropsy was performed the following day, and histopathology was conducted on selected tissues.

## 4. Methods

### 4.1. Sample Preparation

Sterile swabs (HydraFlock^®^, Puritan^®^ Cat. No. 25-3406-H, Guilford, ME, USA) from the abscess or oropharyngeal area were collected on eight occasions between 20 January 2023 and 21 March 2023 to perform next-generation sequencing (NGS). A sterile technique was utilized to avoid contamination, and swabs were immediately stored in sterile tubes containing a DNA preservative buffer (DNA/RNA Shield TM, Zymo Research Corp. Cat. No. R1108, Irvine, CA, USA). Preserved samples were shipped to MiDOG Animal Diagnostics LLC (Tustin, CA, USA), where they were processed and sequenced using methods as described previously [22].

### 4.2. Statistical Analysis

All analyses were conducted in R (Version 4.3.1, R Core Team, 2023). All microbial analyses were conducted using a phyloseq object generated in R Studio (v.2023.06.1-524) from amplicon sequence variance (ASV) data (vegan v2.6.10 [23] and phyloseq v1.50.0 [24] packages). Bacterial diversity was measured using Shannon’s Alpha Diversity Index. To identify the most abundant bacterial species, the ASVs from the phyloseq object were converted to mean relative abundances of each taxa, ordered hierarchically by relative concentration, and plotted as a stacked bar plot for visualization (microshades v1.13 [25], forcats v1.0.0 [26], and ggplot2 v4.0.1 [27] packages).

## 5. Results

### 5.1. Next-Generation DNA Sequencing Results

**Figure 2:**
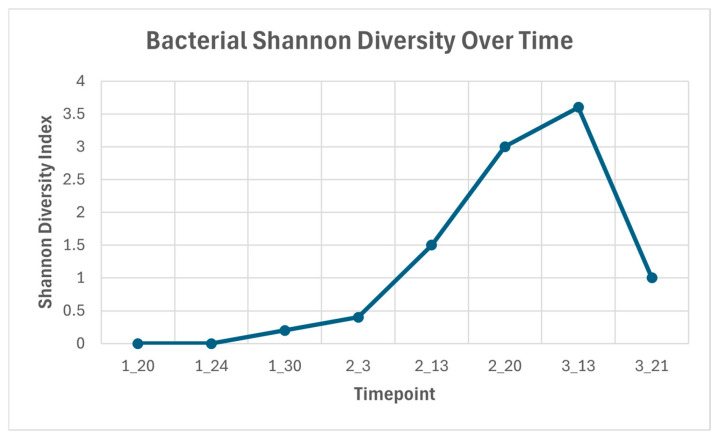
Shannon’s Alpha Diversity Index across timepoints.

The initial NGS results showed an overabundance of *Fusobacterium gonidiaformans*, at 4,900,000 absolute cell counts. Following treatment, subsequent NGS results between 24 January 2023 and 20 February 2023 revealed a 100,000-fold decrease in *F. gonidiaformans*, from 4,900,000 cells to 49 cells. However, despite the clear reduction in this bacterial pathogen, four other bacterial species emerged on 15 March 2023, nearly one month after the cessation of antibiotic treatment. The diversity of the microbial population shifted over time following treatment. As the dominance of *F. gonidiaformans* diminished, the richness and evenness of the entire bacterial composition increased with the emergence of new bacterial species into the sampled area. The relative richness and evenness of bacteria present increased up to timepoint 7, 13 March 2023, with diversity then decreasing at the final timepoint (Figure 2).

**Figure 3:**
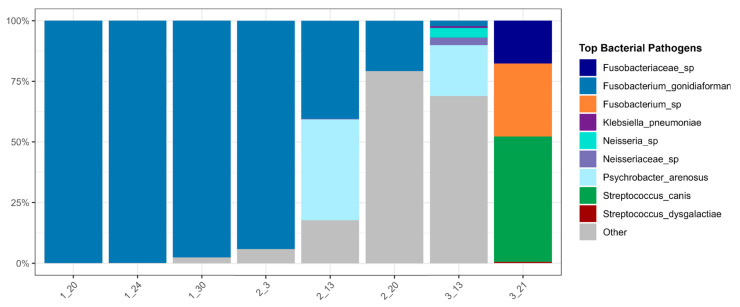
Changes in bacterial pathogens over time. Other indicates non-pathogenic, commensals.

Figure 3 highlights the specific bacterial changes over time. The primary pathogen, *F. gonidiaformans*, is shown to predominate the microbial composition over the first four timepoints (Figure 3). Approximately one month after treatment cessation, the relative abundance of *F. gonidiaformans* dropped, and *Psychrobacter arenosus* was identified (timepoint 5; 13 February 2023), comprising 41.64% of the mean relative abundance of the bacterial composition (Table 1). The emergence of new bacterial species coincided with the increase in relative diversity in the sample (Figure 2), suggesting a shift in species presence and abundance in the microbial composition. At timepoint 7, several new pathogens emerged (Figure 3). Two unidentified species were present within the *Neisseria* genus (3.98% mean relative concentration) and the Neisseriaceae family, at 3.19%. *Klebsiella pneumoniae* is another bacterium that emerged at timepoint 7, comprising 0.64% of the bacterial microbiota (Table 1).

### 5.2. Cytology and Culture Results

Fluid was collected from three sites within the abscess on 9 February 2023, which revealed low to moderate cellularity, mild inflammation, necrotic debris, and low numbers of squamous epithelial cells with mild atypia and no overt evidence of neoplasia or infectious organisms seen. A second sample from 16 February 2023 was suggestive of epithelial neoplasia, but due to cell fragility and degeneration a definitive cytological diagnosis of malignancy was not made. On 16 February 2023 a third sample revealed copious inflammation without evidence of underlying neoplasia and no etiologic agents. On 17 March 2023, cytology and laboratory aerobic and anaerobic culture were conducted (IDEXX). The culture test revealed the presence of a non-descript beta-hemolytic *Streptococcus* taxon and a presumptive, penicillin-resistant *Bacteroides sp*. However, it was unclear whether these taxa were pathogenic or commensal, since species-level identification was not completed.

### 5.3. Histopathology Results

Postmortem tissue samples from the oral cavity identified an invasive squamous cell carcinoma. Multiple specimens from the neck abscess and a cervical lymph node also contained scattered foci of squamous cell carcinoma and abundant necrotic cellular debris. Lung tissue indicated marked necrotizing and hemorrhagic bacterial pneumonia with coccobacilli. The cause of death was attributed to occlusion of the airway due to the oropharyngeal squamous cell carcinoma, aspiration and septic bronchopneumonia. Neoplastic cells were not identified in tissues distal to the oropharynx and neck region. Histopathology on numerous tissues throughout the body indicated the presence of postmortem bacterial overgrowth which precluded further diagnostic testing utilizing NGS.

## 6. Discussion

Complex bacterial infections in wildlife can be challenging to manage and treat due to limitations in the identification of disease-contributing pathogens and effective antimicrobials. Next-generation DNA sequencing provides untargeted screening of the microbial population and antimicrobial resistances for clinical interpretation and treatment assessment by the clinician. Limited studies show the utility of NGS in wildlife infection management, and here we describe one case study where NGS was utilized to longitudinally monitor the microbial composition of a sea lion abscess.

By using NGS, it was possible to detect *F. gonidiaformans* in a sea lion abscess for the first time. The use of NGS afforded the ability to not only detect a reduction in *F. gonidiaformans* over time but also investigate the comprehensive bacterial landscape (Table 1, Figure 3). These analyses revealed an increase in the prevalence of other potential bacterial pathogens that may impact the skin microbiota, including *Streptococcus canis*, *S. dysgalactiae*, *Klebsiella pneumoniae*, and several species within the *Neisseria* and *Fusobacterium* taxonomy. *Neisseria* organisms have typically been seen in individuals with meningitis [28,29] and those with skin lesions [28,30,31]. Thus, while it is possible that the emergence of these species may lead to the propagation of future abscesses, symptoms resolved, and this does not appear to be the case. However, these findings substantiate the rationale to continue microbial surveillance in the long term to ensure that no reemergence of disease occurs. Further, *K. pneumoniae* has previously been considered particularly infectious in marine mammals [32,33,34], having been identified in sea lion blood and skin, typically in those with septicemia, virulent *Klebsiella pneumoniae* infection, and mortality [35,36,37,38]. It is possible that this bacterium was acquired via transmission from another marine mammal, as sea otters have been shown to transfer *K. pneumoniae* to other coastal marine mammals in the American Pacific [39,40]. However, it is not possible to confirm this hypothesis from the available data.

Shortly after the final aerobic and anaerobic cultures were submitted, one more NGS test was conducted on 21 March 2023 that was able to provide species-level identification. *Streptococcus canis* (51.64% relative abundance), *Streptococcus dysgalactiae* (0.44% relative abundance), and an unidentified *Fusobacterium* species (17.67% relative abundance) simultaneously emerged. *S. dysgalactiae* has been shown to contribute to abscess formation in individuals with gastric and nasopharyngeal cancers [41]. In addition, three specific *Bacteroides* species were identified: *B. fragilis* (1.67% relative abundance), *B. stercoris* (0.42% relative abundance), and *B. thetaiotaomicron* (0.64% relative abundance). *B. fragilis* has been previously suggested to be associated with squamous cell carcinoma [42]; thus, while it was found at a lower abundance compared to other bacteria, including *S. dysgalactiae*, it is speculated this bacterium may have contributed to disease progression.

While culture detected the presence of *Streptococcus* generally, NGS more accurately detected specific *Streptococcus* species that have previously been attributed to necrosis. The detection of these microorganisms in both NGS and culture following treatment points to the plausibility that, while treatment effectively eliminated one pathogen (*F. gonidiaformans*) from the abscess, additional pathogens emerged. Unlike culture and cytology, which are limited by the need for viable organisms and often overlook fastidious or slow-growing species, NGS provided a more comprehensive view of the microbial community. In this case, NGS detected additional bacterial taxa not identified by culture. The presence of low numbers of bacteria or fungi in an NGS sample is insignificant in hindsight and in the context of clinical outcomes. During treatment, a low cell count could identify a bacterial or fungal species that is not clinically significant at that time; however, its presence alerts the clinician to its potential growth. This information allows the clinician the opportunity to interpret bacterial abundance in the context of the patient’s response to therapy. Elimination of one bacterium may increase growth opportunities for another. In this case, the rapid increase in the Gram-positive aerobic species *Streptococcus* spp. would have dictated a change in antimicrobial therapy had the patient survived. It also demonstrates that the environment for growth in this abscess transitioned from an anaerobic one to an aerobic one, likely due to more thorough debridement. Longitudinal testing using NGS allows the clinician a comprehensive picture of the microbial composition to be interpreted in the context of clinical treatments and outcomes.

Further identification of microorganisms in NGS beyond that in culture results is important for the clinical context but is subject to interpretation of clinical relevance by the clinician. For example, *P. arenosus* was detected at timepoint 5 at 41.64% of the mean relative abundance of the bacterial composition and suspected to be a putative exogenous organism from the environment that does not impact the skin microbiota. Given that *P. arenosus* virtually disappeared at timepoint 6 and re-emerged at timepoint 7 at a relatively lower percentage, this bacterium is suspected to be a transient colonizer. *P. arenosus* has mostly been considered a non-pathogenic environmental microorganism of the cold-water microbial environment [43]. In the rare cases that *P. arenosus* has been seen in other cold-water marine mammals, such as penguins, it has acted commensally [44]. However, it is important to note that *P. arenosus* has been shown to contribute to blood bacteremia in humans [45]; thus, it may contribute to future infection. Ultimately, the organism was not suspected to contribute significantly to the clinical presentation of this particular patient, but more evidence of its clinical significance is warranted.

For this patient, an underlying neoplastic etiology in the oropharynx and additional bacterial abscesses identified in the lungs were likely the cause of his demise. However, repeated sampling of the abscess helped characterize the changing microbial environment and inform the effectiveness of antibiotic use. *F. gonidiaformans* has been shown to be resistant to erythromycin but sensitive to beta-lactam antibiotics and clindamycin [46]. Thus, this treatment plan was appropriate and appeared to be effective at reducing *F. gonidiaformans*. Anticipation of long-term treatment with antibiotics should be accompanied by repeated testing when possible to ensure the appropriate and judicious use of antibiotics.

## 7. Conclusions

In this case report, Next-generation DNA sequencing (NGS) was used to longitudinally monitor the bacterial composition of a sea lion abscess. NGS provided detection of *F. gonidiaformans* in this cervical abscess, which had previously not been reported in a sea lion (*Zalophus californianus*). While the patient ultimately succumbed to an underlying squamous cell carcinoma of the oropharynx and neck, repeat testing of the abscess provided insight into microbial shifts over time with antimicrobial treatment. Long-term treatment with antibiotics accompanied by repeat testing can support more informed and judicious antibiotic use to the clinician.

## Figures and Tables

**Table 1:** Changes in bacterial pathogens over time as relative percentage of bacterial population.

Species	20 January 2023	24 January 2023	30 January 2023	3 February 2023	13 February 2023	20 February 2023	13 March 2023	21 March 2023
*Bacteroides fragilis*	0.00%	0.00%	0.00%	0.00%	0.00%	0.00%	1.67%	0.00%
*Bacteroides stercoris*	0.00%	0.00%	0.00%	0.00%	0.00%	0.00%	0.42%	0.00%
*Bacteroides thetaiotaomicron*	0.00%	0.00%	0.00%	0.00%	0.00%	0.00%	0.64%	0.00%
*Fusobacteriaceae* sp.	0.00%	0.00%	0.00%	0.00%	0.00%	0.00%	0.00%	17.67%
*Fusobacterium gonidiaformans*	99.92%	99.87%	97.57%	94.05%	40.34%	20.68%	2.31%	0.00%
*Klebsiella pneumoniae*	0.00%	0.00%	0.00%	0.00%	0.00%	0.00%	0.64%	0.00%
*Neisseria* sp.	0.00%	0.00%	0.00%	0.00%	0.00%	0.00%	3.98%	0.00%
*Neisseriaceae* sp.	0.00%	0.00%	0.00%	0.00%	0.28%	0.00%	3.19%	0.00%
*Psychrobacter arenosus*	0.00%	0.00%	0.00%	0.00%	41.64%	0.00%	20.99%	0.00%
*Streptococcus canis*	0.00%	0.00%	0.00%	0.00%	0.00%	0.00%	0.00%	51.64%
*Streptococcus dysgalactiae*	0.00%	0.00%	0.00%	0.00%	0.00%	0.00%	0.00%	0.44%

## Data Availability

The original datasets presented in this study are included in this article; further inquiries can be directed to the corresponding author.
